# Advocacy, experience sharing and action planning toward raising additional financing for primary health care: spending more and spending better towards universal health coverage

**DOI:** 10.1186/s12919-024-00290-5

**Published:** 2024-05-23

**Authors:** Diana Kizza, Juliet Nabyonga-Orem, Brendan Kwesiga, Maria Muniz, Ogochukwu Chukwujekwu, Paul Ngwakum

**Affiliations:** 1 United Nations Childrens Fund, East and Southern Africa Regional Office, ESARO, Nairobi, Kenya; 2 World Health Organization, Regional Office for Africa, Brazaville, Congo; 3 Centre for Health Professions Education, Faculty of Health Sciences, North-West University, Potchefstroom, South Africa

**Keywords:** Health financing, Primary Health Care (PHC), Advocacy

## Abstract

**Background:**

Reallocation of funding to respond the covid-19 pandemic, against a backdrop of longstanding underfunded health systems and high out of pocket expenditures for health, affected access to health services for households, especially those without social protection. These highlighted the urgency in curbing the impact of disruptions on progress towards Universal Health Coverage (UHC) goals. Strategic investments in Primary Health Care (PHC) can help spur the necessary momentum.

**Methods:**

Under the collaborative platform of the Harmonization for Health in Africa’s Health Financing Technical Working Group; UNICEF Regional Office for East and Southern Africa and WHO Regional Office for Africa convened the first PHC financing forum for 21 countries across the Eastern and Southern Africa Region. The three-day forum engaged key health and financing decision makers in constructive dialogue to identify practical actions and policy changes needed to accelerate delivery of UHC through improvements in PHC financing mechanisms and arrangements. The forum was attended by over 130 senior policy makers and technicians from governments, United Nations agencies and nonstate actors drawn from within country, regional and affiliating headquarter institutions.

**Results:**

The Regional Forum engaged participants in meaningful, and constructive discussions. Five themes emerged (1) regular measurement and monitoring of PHC services and spending (2) increasing investments in PHC (3) enhancing efficiency, effectiveness, and equity of PHC spending, (4) ensuring an enabling environment to invest more and better in PHC, and (5) better partnerships for the realization of commitments. An outcome statement summarizing the main recommendations of the meeting was approved at the end of the forum, and action plans were developed by 14 government delegations to improve PHC financing within country-specific context and priorities.

**Conclusions and recommendations:**

The aims of this meeting in augmenting the political will created through the Africa Leadership Meeting (ALM), by catalyzing technical direction for increased momentum for improved health financing across all African countries was achieved. Peer exchanges offered practical approaches countries can take to improve health financing in ways that are suited to regional context providing a channel for incremental improvements to health outcomes in the countries.

## Introduction

The severe economic consequences from the 2020 coronavirus pandemic, climate change, and protracted conflicts have disrupted livelihoods across Sub-Saharan Africa [1]. Responses to such crises have been characterized by a growing emphasis on increasing and reallocating funding towards humanitarian interventions at the expense of planned development efforts [2]. The African region was impacted by the emergency reallocation of funds to respond to the COVID-19 pandemic, which affected continuity and access to primary health services [3], for communities and families, particularly those without social protection, while grappling with severe financial shocks from the unplanned costs of illnesses and fast-rising out of pocket expenditures for health [4]. The pandemic also exposed gaps in the resilience of health systems across Sub-Saharan Africa, with 56% of essential healthcare services, including child and maternal nutrition and health services, HIV treatment, and surgeries having been disrupted due to Covid-19 restrictions and the required resource shifts [5]. This highlighted the urgency in curbing the impact of these disruptions on progress towards Universal Health Coverage (UHC) goals and the need for better health security.

Prioritizing and investing in PHC has long been identified as one of the most effective and affordable channels to achieve better health outcomes and reduce tertiary care costs and overall cost containment for health systems. In the 1978 Alma-Ata Declaration at the International Conference, Primary Health Care (PHC) was identified as the key to the attainment of the “Health for All goal” globally [6]. Later in 2018, the international community reaffirmed its resolve and commitment to PHC in the Declaration of Astana [7]. Improving PHC is a critical enabler of better health system performance and essential foundation for Universal Health Coverage (UHC) and Health Security.

Strategic investments in PHC systems provide a momentous opportunity to reposition the African region on the right trajectory towards 2030 UHC goals.

The effects of COVID-19 pandemic included a significant global acceptance to increase investments in health. This provides an opportune moment through concerted collaboration efforts to advocate for, identify and guide investments to address systemic gaps for PHC delivery and outcome. The question we sought to address is how countries raise additional resources, and how best to optimize existing resources.

The 2022 Lancet Commission on Financing Primary Health Care presented evidence on the levels and patterns of global expenditure on PHC, with an accompanying analysis of the key challenges, and promising practices for countries to consider as actionable policies for low and middle-income countries [8]. Building on this and previous efforts by WHO Council on the Economics of Health for All, World Bank and OECD [9–11]. Beyond issues of financing arrangements for PHC, there is also renewed momentum in operationalizing the measurement of all dimensions for PHC to drive actions for improvement at country level [12]. While there is growing acknowledgment of what needs to be done to move the direction in financing PHC better, there is a need for the discussion to have the countries that implement these initiatives of financing PHC at the center.

The East and Southern Africa Regional (ESAR) PHC Financing Forum was convened to further the discussion on how countries can take forward the learnings on emerging evidence to set out a vision of how to place people at the center of PHC financing.

The PHC Financing Forum provided an accessible opportunity for participants across ESA to convene, and exchange knowledge on how best to translate this information through contextualized peer learnings, applications, and practices on how to fill the PHC funding gaps while building resilient health systems. By developing country-specific roadmaps, it provided an opportunity for governments to convene and collate partner efforts towards catalyzing the required transformations into improve financing for PHC.

## Meeting objectives

The meeting provided a timely opportunity to convene key actors across 21 African countries in ESA, from health and financing sectors to share knowledge and facilitate constructive dialogue to identify practical actions and policy changes to accelerate improvements in PHC Financing. In convening a network of regional PHC Financing partners, this meetings’ specific objectives were to:

- Engage in robust dialogue to share the latest scientific knowledge and country experiences in developing, implementing, and advancing health financing for PHC.
- Agree on way forward regarding a framework on PHC, how to measure PHC progress and PHC financing for increased visibility and efforts to achieve UHC.
- Develop clear action plans for improvement in policies or reforms towards which partners can rally support for increased PHC Financing
- Document outcomes for follow up conversations to link with other ongoing decisions at the African Region meant to strengthen health financing

### Participation

The forum brought together 130 high-level representatives from 20 countries in the region. These included representatives from: Ministry of Health responsible for design and implementation of health financing strategies/reforms and, counterparts from Ministry of Finance especially those responsible for the health desk and representatives from agencies responsible for Social Protection including health insurance. In terms of country representation, it is important to note that a number of countries had senior level representation including the Ministers of Health and Permanent Secretaries for health.

The forum also had convened technical and financial partners working within the region such as Africa Union, Africa CDC, African Development Bank, Bill & Melinda Gates Foundation, Clinton Health Access Initiative, Global Financing Facility, Global Fund, Public Health Institute, UNICEF, UNFPA, WHO and USAID. There was representation from Civil Society Organizations such as PATH, IBP and research/academic institutions such as KEMRI Welcome Trust, London School of Hygiene and Tropical Medicine, and Rwanda School of Public Health.

## Methods

Under the collaborative platform of the Harmonization for Health in Africa’s (HHA) Health Financing Technical Working Group; UNICEF and WHO AFRO convened the first Primary Health Care financing forum for 21 countries across Eastern and Southern Africa Region. The three-day forum was a deliberative workshop that focused on engaging key health and financing sector decision makers, to facilitate constructive dialogue to identify practical actions and policy changes to accelerate delivery of Universal Health Coverage through improvements in PHC Financing mechanisms and arrangements.

Employing curated technical presentations, moderated panels, and public discussion, the forum was organized into three main sessions that explored comprehensive financing drivers of resilient primary health care delivery. Day one focused on defining PHC and an in-depth discussion of existing operational and monitoring frameworks. Day two dived into the levers for additional domestic resource mobilization including the re-allocation of finances towards PHC, Health Taxes, Pooling resources for PHC, the role of the private sector in financing PHC, and the opportunity to leverage the evolving donor financing. Day three, focused on how to ensure finances are allocated and used efficiently to promote effective PHC delivery for UHC, including topics on strategic purchasing, provider payment mechanisms, re-allocation and optimal health financing arrangements, mechanisms, and systems.

Moderated panel sessions were curated to enable a deep dive on the practices by different countries on the three core areas, highlighting their challenges, and how they overcame them. The topics on levers for domestic resource mobilization, equitable allocation through pooling and incentives for efficiency through strategic purchasing of health services were presented through practical case studies from member countries. This provided strong learning for other like-minded countries in building replication strategies.

## Results

The meeting resulted in five emerging themes on (1) regular measurement and monitoring of PHC services and spending (2) increasing resources to invest in PHC services, and (3) enhancing efficiency, effectiveness, and equity of PHC spending, (4) the enabling environment for investing more and better in PHC services, and (5) strengthening partnerships to enable better implementation. By the end of the forum an outcome statement summarizing the main recommendations of the meeting was approved, and action plans were developed by 14 government delegations to improve PHC financing within country-specific context and priorities.

The published outcome statement [13] summarizes the main recommendations from the Primary Health Care (PHC) Financing Forum for Eastern and Southern Africa (ESA) held on 21–23 March 2023 in Kigali, Rwanda through the Harmonization for Health in Africa (HHA) platform.

The forum provided a platform for an ultimate objective of finding ways to ensure increased and equitable access to quality and comprehensive health care services. Importantly, by the end of the workshop, each government delegation developed an action plan to improve PHC financing reflecting country-specific contexts and priorities. These country specific action plans will be further discussed, revised, and validated by the relevant government officials and partners within each country, with the intention of rallying global partner support to accelerate efforts to invest in PHC as a key strategy for achieving Universal Health Coverage.

A summary of the key discussions and recommendations agreed upon to improve PHC financing and spending are presented below.

### Regular measurement and monitoring of PHC services and spending

Sustainable investments in PHC starts with a shared understanding of the meaning and functional boundaries of PHC. This will help in prioritizing, optimizing and sequencing PHC interventions. Delegates agreed that comprehensive and regular monitoring and measurement of PHC services, financial inflows, and expenditures by each government at all levels of care, including at the community level, is critical.

#### Key recommendations

- Facilitate the development of national consensus, using a whole of government and whole of society approaches on the national definition as well as functional and programmatic boundaries of PHC to guide planning, budgeting, resource mobilization, and expenditures.
- Ensure evidence-based decision-making, develop robust mechanisms for tracking financial inflows, allocations, and expenditures on PHC based on national definitions while drawing guidance from international approaches and good practices. This may include elaboration of the PHC expenditures as part of national health accounts, or the use of programme-based budgeting or budget tagging.
- Make timely information about PHC financing and expenditures publicly available to improve transparency, accountability, and public participation in decision-making.
- Strengthen national and local data and information systems as well as quality assurance, monitoring and evaluation of PHC plans and budgets.

### Increasing resources to invest in PHC services

Domestic public revenue is the most sustainable way to finance the provision of quality PHC services for all. Strengthening domestic revenue mobilization for the health sector including during emergencies and other shocks, as well as strategic allocation of available resources will continue to be a policy priority of governments in Eastern and Southern Africa. However, considering fiscal space constraints and recurring shocks, external financing may be used as supplementary funding especially in low and lower-middle income countries where government revenue is limited, debt distress is rising, and macro-economic volatility is high.

#### Key recommendations

- Facilitate costing of a nationally defined PHC package and other health sector interventions to inform resource mobilization and annual budgeting.
- Recommit to increasing public investments in PHC to match estimated need as per the costed plans and to meet the 2001 Abuja Declaration for governments to allocate at least 15 per cent of their budgets to health.
- Strengthen the engagement of PHC stakeholders including local authorities, civil society organizations, and private sector in policy processes related to revenue raising, pooling and strategic purchasing of health services in general and PHC.
- Develop policies to reduce fragmentation across resource pools in the health sector and ensure pooled funds are directed towards PHC.
- Crowd in domestic public financing including through progressive taxation, public-private partnerships for health and where feasible through mechanisms such as taxes on alcohol, tobacco, and sugar-sweetened beverages and earmarking the funds for health where such mechanisms do not risk reduction in other sources of revenue.
- Design robust PHC financing mechanisms such as provider payment schemes, capitation, performance/ results-based financing, and direct facility financing so that resources are channeled to the most marginalized and hard-to-reach areas.
- Strengthen mechanisms for financing PHC services during emergencies considering recurrence of shocks such as health emergencies, droughts, cyclones, and conflict.
- Explore external financing opportunities including but not limited to concessional loans, grants, Special Drawing Rights (SDRs), and debt-for-health swaps as supplementary financing. Such external funding should be aligned with the national health priorities, delivered through pooled funding mechanisms, and made on-budget as much as possible.
- Wherever possible and as needed conduct cost-benefit and other relevant analyses to demonstrate the impact of PHC investment on the health system and the economy.

### Enhancing efficiency, effectiveness, and equity of PHC spending

Sustainable investment in PHC is not just a question of more inflows, it is also about better use of all available domestic and external resources. By better emphasizing efficiency, value for money and equity in PHC spending, there is significant scope to increase coverage, quality, and impact of resources especially on the most vulnerable populations.

#### Key recommendations

- Explore opportunities created by budgeting approaches such as programme/ output/ performance-based budgeting to improve allocative efficiency and ensure all elements of the PHC framework receive a fair share of available resources.
- Strengthen measures to ensure that the health workers needed to support PHC are available, with the required capacity and are well motivated. This should include community health workers who help in the delivery of PHC especially in underserved communities.
- Address bottlenecks that limit timely flow of funds to service delivery points and optimize relevant structures to ensure timely and efficient disbursement of funds.
- Periodically assess PHC budget implementation bottlenecks, especially in supply and procurement, and take measures to improve budget credibility and execution.
- Strengthen mechanisms for strategic purchasing including appropriate mix of provider payment mechanisms.
- Review or strengthen intergovernmental fiscal transfer mechanisms in decentralized settings and use health resource allocation formulae to improve equity in resource allocation within countries.
- Strengthen health sector governance systems for improved accountability, transparency, and public participation in PHC financing and spending at national, sub-national and local levels.
- Ensure other sectors like water, sanitation, education, and agriculture, which contribute to quality PHC get a fair share of available resources.
- Institutionalize mechanisms for bringing more value for the money invested in health, including regular diagnostics to assess and improve cost-efficiency and effectiveness in procurement and delivery of PHC services.

### The enabling environment for investing more and better in PHC services

Comprehensive national and health sector policies and plans that put the right level of priority on PHC as well supportive institutional frameworks underpinned by robust public financial management (PFM) systems are key enablers for improved funding, budgeting, and spending on PHC services.

#### Key recommendations

- Regularly update national and health sector policies and plans to ensure they are oriented towards PHC, and they are costed to inform annual budgets and medium-term expenditure frameworks.
- Strengthen sub-national health systems, including on data and information, planning, forecasting, and costing, supply, and procurement, as well as reporting and accountability to enable last mile delivery.
- Strengthen partnerships and harmonization for PHC comprehensive approach, including measures for the strategic involvement of the private sector in PHC, including through enhancing the capacities of relevant ministries and departments to design, regulate, monitor, and evaluate public private partnership on health, and create clear and sustainable incentives to attract private investments in PHC.
- Foster a whole of government and whole of society approaches to PHC planning, budgeting and service delivery including through national multi-stakeholder dialogues and co-creation of interventions.
- Seize opportunities offered by regional and global institutions to share insights and lessons learned and continue advocating for improved public investment in PHC.

## Discussion and conclusion

As a region, we have strong political will to see to it that all resources are availed to ensure our communities have equitable access to quality PHC services. The real issue is where will the additional resources come from given competing demands and priorities stemming from complexities from related mitigation of conflict and public health emergencies.

Through ongoing efforts from the AU Regional Economic Centre’s on the ALM Agenda, strong political will for the agenda on increased health financing has been established. The challenge of fiscal space for health, however, is where to obtain the additional resources required to achieve the intended health SDG and UHC goals.

Peer exchange on ways to increase efficiencies of existing resources, identify practical financing arrangements and domestic mechanisms that work within the regional context; provide a channel for incremental and catalytic improvements across themes on Public Financial Management, provider incentives, and strategic partnerships using existing systems. There is indeed room to improve efficiency in health spending as reported by Arhin et al., and available resources can improve the UHC score of countries by at least 19% [14]. Through regular convenings that challenge individual country status quo thinking an impetus for improvements and peer reflections on where to source additional resources for health can be built. This convening played a significant role in the quality of actions for feedback into the Africa Head of States annual ALM convenings and tracker, which provide the political level platform upon which additional change can be leveraged for the required adaptations and improvements required to advance the goals of the ALM as observed through the ALM tracker.

## Conclusion

In building momentum for progressive change across all the countries in the African region, this convening challenged individual country status quo thinking, providing impetus for improvements born of peer reflections on where to source additional resources for health. This is critical to move forward the Africa Leadership declaration on improving financing for health in Africa and hence is an opportunity to operationalize the political platform upon which additional change can be sought or approved for the required adaptations and improvements required to advance the goals.
